# Cerebellar Ataxia, Impaired Intellectual Development, and Disequilibrium Syndrome-2: A Case Report

**DOI:** 10.7759/cureus.78066

**Published:** 2025-01-27

**Authors:** Livia Hochman, Alrick Drummond, Kara Morgan

**Affiliations:** 1 Medicine, Florida State University College of Medicine, Tallahassee, USA; 2 Pediatrics, Florida State University College of Medicine, Tallahassee, USA; 3 Genetics, University of South Florida Health, Tampa, USA

**Keywords:** camrq2, cerebellar ataxia, disequilibrium syndrome, impaired intellectual development, wdr81

## Abstract

Pathogenic variants in the *WDR81* gene on chromosome 17p13.3 have been linked to cerebellar ataxia, impaired intellectual development, and disequilibrium syndrome-2 (CAMRQ2), a rare disorder characterized by congenital cerebellar ataxia (a condition causing impaired coordination and balance due to cerebellar dysfunction), intellectual disability, and gait abnormalities. Additional features include thoracic kyphosis, scoliosis, short stature, intention tremor, and cerebellar atrophy. We present a case of a mildly affected female from a non-consanguineous family, expanding the clinical spectrum of this disorder. The patient, born at term as part of a dizygotic-diamniotic twin pregnancy, exhibited developmental delays, feeding difficulties, and unsteady gait. This case highlights the importance of iterative genetic testing, as initial evaluations, including brain MRI and genetic testing, were nondiagnostic. However, reanalysis at age five identified a homozygous pathogenic variant in *WDR81*, demonstrating how periodic re-evaluation of genetic data can aid in diagnosing rare disorders that may have been previously unrecognized. The patient continues to experience cerebellar ataxia and hypotonia, characterized by decreased muscle tone and reduced strength, with no other major medical conditions. She receives physical and occupational therapies and is academically at grade level with tutoring support. This case highlights the phenotypic variability of CAMRQ2 and underscores the importance of considering *WDR81* variants in patients with cerebellar ataxia, even in the absence of consanguinity.

## Introduction

Pathogenic variants in the *WDR81* gene located on chromosome 17p13.3 are associated with cerebellar ataxia, impaired intellectual development, and disequilibrium syndrome-2 (CAMRQ2). This disorder is characterized by congenital cerebellar ataxia, intellectual disability, and gait abnormalities [1]. This disorder is extremely rare, with fewer than 20 previously published cases in the literature [2]. All cases of CAMRQ2 that have been reported are consanguineous with autosomal recessive inheritance [3,4]. The *WDR81* gene has been found to show some similarity with genes including NSMAF (neutral sphingomyelinase activation associated factor), NBEA (neurobeachin), and LYST (lysosomal trafficking regulator) [1,5,6,7]. Previously published cases describe other varying features, including thoracic kyphosis and scoliosis, short stature, absent or limited speech, intention tremor, coarse facial features, hirsutism, strabismus, wide and short neck, and small hands and feet [2]. Findings on brain MRI of previously published cases include cerebellar atrophy, generalized brain atrophy, and hypoplasia of the corpus callosum [2]. All previously published cases have occurred in consanguineous families, and the majority of affected individuals ambulated with a quadrupedal gait [2]. Here, we describe a case report of a more mildly affected female with bipedal gait from a nonconsanguineous family, expanding the phenotype of this rare disorder.

## Case presentation

**Figure 1:**
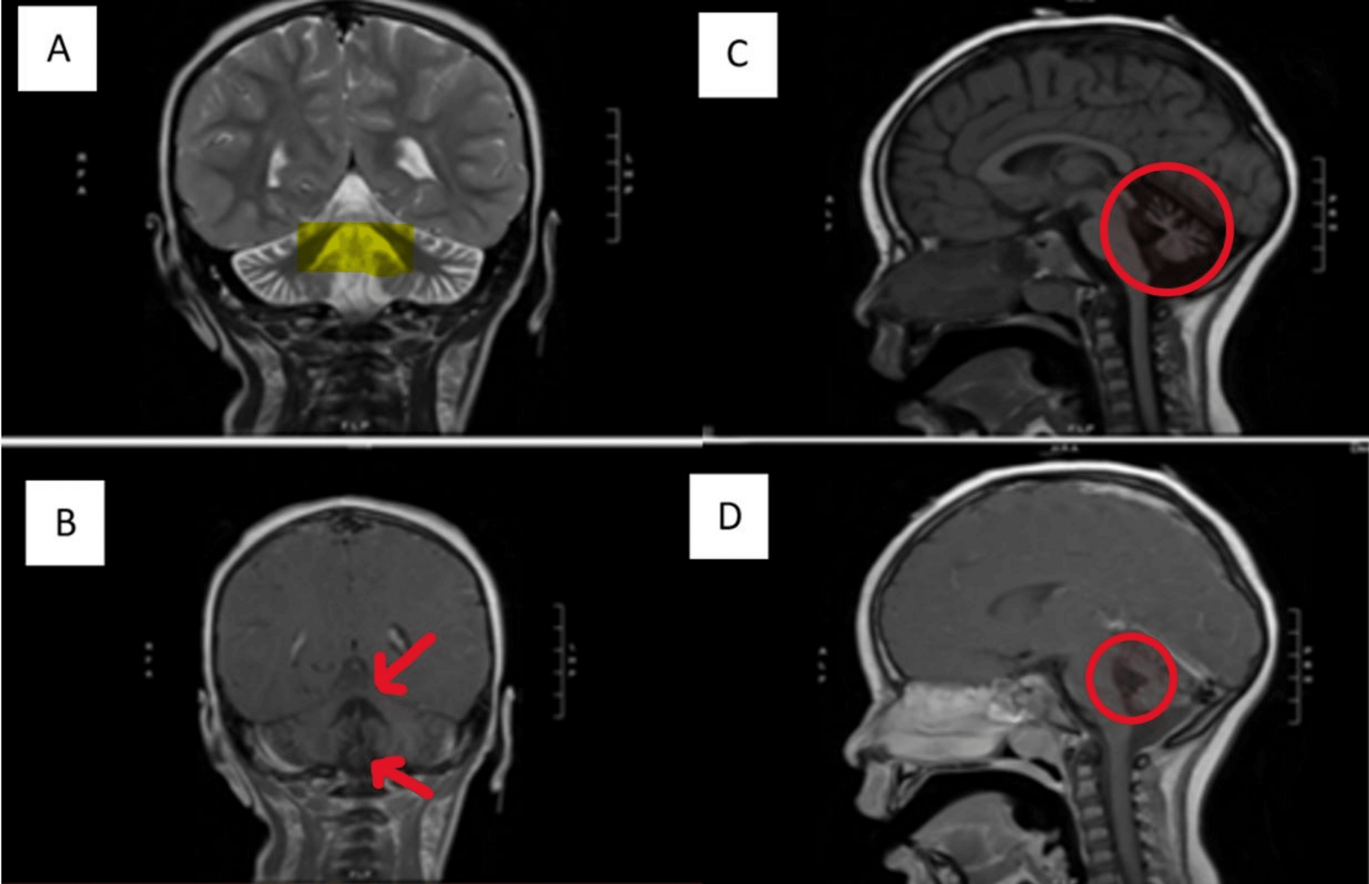
Brain MRI findings Brain MRI shows cerebellar atrophy, most pronounced in the vermis, with ex-vacuo dilation of the fourth ventricle. Figure A presents the coronal T2 view. The yellow highlighted area shows an enlarged fourth ventricle. Figure B presents coronal T1 post-contrast view. The red arrows depict atrophy of the superior and inferior vermis. Figure C presents sagittal T1 FLAIR view. As shown in this red circle, the cerebellum does fully occupy its allotted space. Figure D presents sagittal T1 post-contrast view. The fourth ventricle is dilated, as evidenced by the increased hypointense area within the red circle.

The patient was twin A of a dizygotic-diamniotic pregnancy, born at 37 6/7 weeks to a 26-year-old G1P0 mother who received regular prenatal care. She was transferred to the neonatal intensive care unit (NICU) due to hypoglycemia and vomiting. The NICU course included a neurology consult for apneic episodes and concern for subclinical seizures. Head ultrasound reported multiple punctate echogenic foci in the basal ganglia bilaterally, but no structural abnormalities were reported on brain MRI at five days of age. The electroencephalogram (EEG) was normal in the NICU, and she was discharged at 10 days of age. Her family developed concerns for her development around age six months when they noted she was not attaining milestones at the same pace as her fraternal twin. They noted difficulty with feeding and choking episodes, with the patient requiring the use of a level 1 bottle nipple until she transitioned to solid foods. She had difficulty sitting unassisted and would often fall to the side. She began walking at 16-18 months of age but with an unsteady gait and frequent falls. The family also noted a hand tremor when pointing and reaching for objects. She was evaluated by neurology, and at 18 months of age, she was diagnosed with cerebellar ataxia. Brain MRI reported cerebellar atrophy, most pronounced in the vermis, with ex-vacuo dilation of the fourth ventricle (Figure 1).

Following the MRI results, she was evaluated by multiple other subspecialties, and work-up including spine MRI, cardiac evaluation, ophthalmology evaluation, biochemical testing for inborn errors of metabolism, ataxia gene panel, spinal muscular atrophy gene testing, chromosome microarray, and whole exome sequencing trio with mitochondrial DNA analysis were nondiagnostic at that time. The family relocated and the patient was re-referred to a clinical genetics clinic at five years of age. Re-analysis of the data from the previous whole exome trio resulted in a homozygous pathogenic variant in *WDR81*.

The patient continues to experience cerebellar ataxia and frequent falls and has hypotonia and mild nystagmus, but no other medical conditions. She receives physical and occupational therapies. She underwent an evaluation for speech therapy as a preventive measure but did not qualify for services due to her age-appropriate skills. She is working at grade level in school with tutoring support but without formal academic interventional services. Family history is negative for similarly affected individuals and consanguinity was denied.

Results

The whole exome sequencing trio with subsequent data reanalysis identified a homozygous c.2567 C>T [p.(P856L)] variant in exon 1 of the *WDR81* gene. Each parent was found to be heterozygous for this variant, consistent with autosomal recessive inheritance. 

## Discussion

Türkmen et al. (2006), Tan (2006), and Gulsuner et al. (2011) studied a consanguineous Turkish family with five siblings with structural brain abnormalities including cerebellar hypoplasia, severe intellectual disability, limited speech, and quadrupedal ambulation secondary to inability to walk with a bipedal gait [1,2,8]. Genome-wide linkage analysis identified a candidate disease locus on chromosome 17p [2]. Ozcelik et al. (2008) confirmed linkage to chromosome 17p13 in the same family [9]. Moreover, in this same family, Türkmen et al. (2006) discovered a homozygous mutation in the *WDR81* gene (P856L), which segregated from the phenotype and was not present in 549 control subjects [1].* WDR81* was also found to be highly expressed in the cerebellum and corpus callosum [1]. MRI findings presented hypoplasia of the cerebellum and cerebellar vermis, a small nucleus dentatus, and a thin corpus callosum [2].

Alazami et al. (2015) identified a homozygous missense variant in the *WDR81* gene (G282E) in a patient with hydranencephaly and cerebellar hypoplasia, from a consanguineous family, further supporting an autosomal recessive inheritance pattern and the *WDR81* gene’s involvement in brain development [10]. Komara et al. (2016) reported two siblings in a consanguineous Yemeni family with global developmental delay, limited speech, cerebellar hypoplasia, and bipedal ataxic gait [11]. The siblings were found to have a homozygous truncating mutation in the *WDR81* gene (R1333X) by whole-exome sequencing, which was confirmed by Sanger sequencing and segregated with the phenotype in affected individuals [11]. Notably, the bipedal ataxic gait in the siblings suggests that although the quadrupedal gait is a common and significant feature in severe cases of CAMRQ2, a broader spectrum of motor phenotypes can be seen [11]. Genes involved in quadrupedal locomotion include* VLDLR*,* CA8*, and* WDR81.* However, some studies have shown that the *WDR81* gene is not co-expressed with genes such as *VLDLR* and *CA8* [1,12,13]. This raises the possibility that *WDR81* might play a role in a separate developmental regulatory pathway [1].

As described above, published cases of CAMRQ2 are characterized by severe intellectual disability including limited or absent speech, with most affected individuals using quadrupedal ambulation secondary to the inability to walk with a bipedal gait [1,2,8,9,10,11]. Our patient has a notably milder developmental phenotype. She has age-appropriate speech skills and a bipedal gait (although with ataxia and frequent falls), and she is able to jump on two feet and dance. Interestingly, she has the same variant in the *WDR81 *gene (P856L) as in the Turkish family studied [1,2,8]. This case underscores the importance of considering *WDR81* mutations even in the absence of consanguinity and severe intellectual disability. Furthermore, the patient’s relatively preserved cognitive abilities and age-appropriate speech suggest that early therapeutic interventions, along with environmental factors, may play a critical role in mitigating the severity of the disease. We propose that her early diagnosis and therapeutic interventions have contributed to her milder presentation. This case emphasizes the importance of long-term follow-up to assess the full range of possible outcomes in these patients.

## Conclusions

CAMRQ2 is an extremely rare condition, and our patient demonstrates previously undescribed phenotypic variability with cerebellar ataxia but otherwise age-appropriate development. This case highlights the importance of iterative genetic testing and shows how gains in information and technology over time can identify rare diagnoses. In our patient's case, early diagnosis allowed for targeted therapies, such as physical and occupational therapy, which have contributed to a more favorable outcome compared to reported cases of CAMRQ2 presenting with quadrupedal ambulation and severe intellectual disability.

## References

[1] Gulsuner S, Tekinay AB, Doerschner K (2011). Homozygosity mapping and targeted genomic sequencing reveal the gene responsible for cerebellar hypoplasia and quadrupedal locomotion in a consanguineous kindred. Genome Res.

[2] Türkmen S, Demirhan O, Hoffmann K, Diers A, Zimmer C, Sperling K, Mundlos S (2006). Cerebellar hypoplasia and quadrupedal locomotion in humans as a recessive trait mapping to chromosome 17p. J Med Genet.

[3] Kolb LE, Arlier Z, Yalcinkaya C (2010). Novel VLDLR microdeletion identified in two Turkish siblings with pachygyria and pontocerebellar atrophy. Neurogenetics.

[4] Moheb LA, Tzschach A, Garshasbi M (2008). Identification of a nonsense mutation in the very low-density lipoprotein receptor gene (VLDLR) in an Iranian family with dysequilibrium syndrome. Eur J Hum Genet.

[5] Ward DM, Griffiths GM, Stinchcombe JC, Kaplan J (2000). Analysis of the lysosomal storage disease Chediak-Higashi syndrome. Traffic.

[6] Rudelius M, Osanger A, Kohlmann S (2006). A missense mutation in the WD40 domain of murine Lyst is linked to severe progressive Purkinje cell degeneration. Acta Neuropathol.

[7] Karim MA, Suzuki K, Fukai K (2002). Apparent genotype-phenotype correlation in childhood, adolescent, and adult Chediak-Higashi syndrome. Am J Med Genet.

[8] Tan U (2006). A new syndrome with quadrupedal gait, primitive speech, and severe mental retardation as a live model for human evolution. Int J Neurosci.

[9] Ozcelik T, Akarsu N, Uz E (2008). Mutations in the very low-density lipoprotein receptor VLDLR cause cerebellar hypoplasia and quadrupedal locomotion in humans. Proc Natl Acad Sci U S A.

[10] Alazami AM, Patel N, Shamseldin HE (2015). Accelerating novel candidate gene discovery in neurogenetic disorders via whole-exome sequencing of prescreened multiplex consanguineous families. Cell Rep.

[11] Komara M, John A, Suleiman J, Ali BR, Al-Gazali L (2016). Clinical and molecular delineation of dysequilibrium syndrome type 2 and profound sensorineural hearing loss in an inbred Arab family. Am J Med Genet A.

[12] Hartl D, Irmler M, Römer I (2008). Transcriptome and proteome analysis of early embryonic mouse brain development. Proteomics.

[13] Huang da W, Sherman BT, Lempicki RA (2009). Systematic and integrative analysis of large gene lists using DAVID bioinformatics resources. Nat Protoc.

